# Précis on Knowing and Checking: an Epistemological Investigation

**DOI:** 10.1007/s12136-022-00540-1

**Published:** 2022-12-27

**Authors:** Guido Melchior

**Affiliations:** Department of Philosophy, University of Graz, Heinrichstrasse 26/5, 8010 Graz, Austria

## Abstract

In this Précis, I provide an overview of my *Monograph Knowing and Checking: An Epistemological Investigation* (Melchior 2019), which is subject to a book symposium organized by the University of Maribor. This volume in *Acta Analytica* contains contributions by Peter Baumann, Kelly Becker, Marian David, Nenad Miščević, Robert Weston Siscoe, and Danilo Šuster along with my replies.

*Knowing and Checking: An Epistemological Investigation* (Melchior 2019), hereinafter KC, is primarily about checking, and derivatively about knowing. In Part I of KC, I develop a theory about checking. In Part II, I use this theory to explain persistent puzzles about knowledge, focusing specifically on puzzles related to knowledge closure and the skeptical puzzle. Checking is one of the most common epistemic concepts. Nevertheless, checking has been neglected in epistemology. KC aims to fill this oversight by providing a theory about checking. The account of checking that I propose is:

- S *checked that p* was true via method M iff
  1. S intentionally used M for determining whether *p* is true.
  2. M has certain modal features with respect to *p* (especially sensitivity).
  3. M accurately indicated that *p*.

In KC, I argue that the modal features of the method used determine its appropriateness as a method of checking whether *p* is true. In particular, I contend that sensitivity is the crucial modal condition on checking, i.e., one cannot successfully check whether *p* is true by using a method that would indicate that *p* is true if *p* were false. Sensitivity is a controversial condition of knowledge. Thus, sensitivity is necessary for checking but plausibly not for knowing, thereby marking a crucial distinction between knowing and checking.

KC aims to achieve three main goals. First, it contains a theory about checking, which includes internalist components such as the intentions of the checking subject as well as externalist components, in particular, the modal features of the method used. Second, it provides a fresh view on enduring knowledge problems, namely closure problems and the skeptical problem, by explaining intuitions about knowing in terms of intuitions about checking. Third, KC aims at settling a dispute about epistemic modal conditions by finding a new home for the intuitively appealing but nevertheless controversial sensitivity condition as the crucial necessary condition on checking.

KC also follows an alternative methodological path. Most epistemological theories focus on knowledge as the most significant epistemic concept. However, our epistemological vocabulary is much richer than simply talking of knowing, including the concepts of checking, arguing, proving, understanding, or demonstrating. However, many of these other epistemic concepts are rather neglected in epistemology. This is a shortcoming for two reasons. First, a theory of these alternative epistemic concepts is interesting and desirable in its own right. Second, such a theory can be used to provide a deeper understanding of knowledge. Following this strategy, I develop in KC a theory about checking that I then use to explain existing intuitions about knowing. For example, I argue that, in contexts of checking, when we raise the question whether *p* is true and intentionally reflect about a method for settling this question, we think that for knowing a method is required that it is also appropriate for checking. That means that, in contexts of checking, we have the intuition that knowing requires a sensitive method. Let me now provide an overview of KC.

## Chapter 2: Modal Knowledge Accounts

Chapter 2 contains an overview of modal knowledge accounts, focusing on sensitivity. Nozick (1981) provided the following influential modal knowledge account:

- S knows that *p* iff
  1. *p* is true.
  2. S believes that *p*.
  3. In the nearest possible worlds where *p* is false, S does not believe that *p*.
  4. In the nearest possible worlds where *p* is true, S believes that *p*.

Condition (3) has become known as the sensitivity condition and condition (4) as the adherence condition. Nozick argues that this definition can solve the Gettier problem and the skeptical problem, since gettierized subjects do not sensitively believe and our beliefs in anti-skeptical hypotheses also fail to be sensitive. Nozick realized that his knowledge account encounters problems if it does not take the belief-forming method into account and provided the following definition of knowing via a method:

- S knows, via method (or way of believing) M, that *p* iff
  1. *p* is true.
  2. S believes, via method or way of coming to believe M, that *p*.
  3. In the nearest possible worlds where *p* is false and where S uses M to arrive at a belief whether (or not) *p*, S does not believe, via M, that *p*.
  4. In the nearest possible worlds where *p* is true and where S uses M to arrive at a belief whether (or not) *p*, S believes, via M, that *p*.

In cases where S believes via various methods, S knows simpliciter according to Nozick if the dominant method that “outweighs” the other methods fulfills sensitivity and adherence. When analyzing checking, we always consider a specific method. Thus, an analysis similar to Nozick’s concept of knowing via a method will be crucial for a theory of checking.

Nozick’s knowledge account, and in particular its sensitivity condition, quickly became subject to serious criticism. First, beliefs via induction tend to be insensitive, as Vogel (1987) and Sosa (1999) pointed out. Since induction can plausibly yield knowledge, sensitivity cannot be necessary for knowing. Related problems arise for higher-level knowledge, which also tends to be insensitive.^1^ The second objection against sensitivity concerns closure. Nozick’s account of knowledge violates the closure principle for knowledge, i.e., it is possible that S knows that *p*, knows that *p* entails *q*, but still fails to know that *q*. Nozick regarded this feature as advantageous since it allows knowing ordinary propositions without knowing anti-skeptical hypotheses, but as Kripke (2011) pointed out, knowledge also violates closure in highly implausible cases according to Nozick’s sensitivity account. Third, Luper-Foy (1984) showed that one-sided methods that can only indicate that *p* but not that ~ *p* cannot be sensitive and therefore cannot yield knowledge, an implausible consequence.

Despite these problems, the sensitivity principle has remained intuitively appealing, leading to a second wave of sensitivity accounts, as Becker and Black (2012) call, it, which aim at meeting the objections raised against Nozick’s original formulation. DeRose (1995, 2010, and 2017) defends a contextualist knowledge account, according to which sensitivity is necessary for knowing in some more demanding contexts but not necessary in other contexts, preserving knowledge closure in all contexts.^2^ Black (2002) argues against Nozick for characterizing methods externalistically, thereby allowing for sensitive knowledge of anti-skeptical hypotheses. Roush (2005) provides a sensitivity account that incorporates insensitive knowledge via deduction from sensitive knowledge, thereby also preserving knowledge closure. Becker (2007) develops an externalist knowledge account by combining elements of sensitivity and reliabilism. Despite these suggestions, sensitivity remains a controversial condition on knowledge, and safety, as defended by Sosa (1999) and Pritchard (2005), is nowadays the more popular modal knowledge condition.

## Chapter 3: SAC: a Sensitivity Account of Checking

Chapter 3 is the central chapter of KC. I develop there a sensitivity account of checking, SAC. Two conditions on checking whether *p* is true via a method M are specified, (1), that S uses M with the intention of determining whether *p* is true, and, (2), that M is an appropriate method with respect to *p*. The first condition is specified internalistically by the intentions of the checking subject, and the second condition externalistically by the modal profile of the method. Since checking is a process, we can distinguish ex ante reports about checking from ex post reports. In KC (35), I am interested in the conditions of successfully checking. Thus, I focus on ex post reports of checking of the following form:

- S *checked that p* was true via M iff
  1. S intentionally used M for determining whether *p* is true.
  2. M is a checking method with respect to *p*.
  3. M accurately indicates that *p*.

I assume in KC that a subject can check successfully simply by using a checking method but without knowing the modal features of the method used. However, I also define reflective checking as checking that involves this kind of knowledge.

Whether a method is proper for determining whether *p* is true depends on its modal profile, i.e., it depends on what the method would indicate under certain circumstances or in particular possible worlds. Thus, when it comes to checking, we talk about the modal features of *methods* instead of the modal features of beliefs based on methods. Methods can have different modal profiles concerning a particular proposition *p*. In KC, I distinguish various modal features of methods, including sensitivity, adherence, safety, and negative safety, all coming in a weak and a strong version.^3^ Perfect methods for checking have intuitively a simple modal profile: (1) If *p* were true and M were used to determine whether *p*, then M would indicate that *p* is true; and (2) If *p* were false and M were used to determine whether *p*, then M would indicate that *p* is false. However, we also want to allow non-ideal methods as checking methods, for example methods that are asymmetric concerning their indication of *p* and ~ *p*. Nevertheless, we do not allow any method to be adequate for checking. In particular, we want to exclude the following three types methods from being checking methods:

1. Random methods
  M makes random indications about the truth of *p* when used to determine whether *p* is true.
2. Opposing methods
  M always makes false indications concerning *p*.
  If *p* were true and M were used to determine whether *p* is true, then M would indicate that *p* is false and if *p* were false and M were used to determine whether *p* is true, then M would indicate that p is true.^4^
3. Monotonous methods
  M always makes the same indication concerning *p*, regardless of whether *p* is true or false.
  If *p* were true and M were used to determine whether *p* is true, then M would indicate that *p* is true and if *p* were false and M were used to determine whether *p* is true, then M would indicate that *p* is true.

Each of these methods fails to be sensitive, since it is strongly insensitive in the following sense:

- Strong insensitivity
  M is strongly insensitive with respect to *p* iff: If *p* were false and M were used to determine whether *p* is true, then M would indicate that *p* is true.

As I argue in KC, fulfilling sensitivity and avoiding strong insensitivity are the crucial modal features of checking methods. Safety, in contrast, is also necessary, but it is not an explanatorily powerful condition. As I show, methods that are safe but insensitive are not appropriate methods for checking, although they might be regarded as appropriate for acquiring knowledge. Moreover, I discuss in Chapter 3 various modal features of asymmetric methods and whether they are suitable for checking.

Externalist knowledge accounts face the generality problem, i.e., the problem of determining the method used. Since the checking account in KC is externalist in nature, it seems to be also subject to this problem. However, I argue in KC that this is not the case for checking, since the method is specified by the intentions of the checking subject to use a particular method for checking. Notably, this solution is not available for theories of knowing, since we can acquire knowledge unintentionally without intentionally using a particular method. Finally, I reflect in Chapter 3 on Kripke’s barn case against sensitivity, arguing that with checking the violation of closure is less problematic than in the case of knowing.

## Chapter 4: Checking, Alternatives, and Discrimination

In Chapter 3, “Chapter 3: SAC: a Sensitivity Account of Checking”, I develop an account for checking whether *p* (or ~ *p*) is true. I call this form of checking *checking simpliciter*. However, we cannot only check whether *p* is true, but we can also check whether *p* or a particular alternative *q* is true. In Chapter 4, I extend the developed checking account, taking specific alternatives into account. If we consider the case of Peter cleaning the kitchen today, then we can distinguish between checking that *Peter* (and not somebody else) cleaned the kitchen today, checking that Peter cleaned the *kitchen* today (and not something else), and checking that Peter cleaned the kitchen *today* (and not on some other day). I argue that these forms of checking differ along two dimensions. First, checking whether *p* or *q* is true involves different intentions of the checking subject than checking whether *p* is true or checking whether *p* or *r* is true. Second, the sensitivity conditions for the different forms of checking differ. In cases of checking simpliciter, a proper method would not indicate that *p* is true if *p* were false. A proper method for checking whether *p* or *q* is true would not indicate that *p* is true if *q* instead of *p* were true. As I argue, these different sensitivity conditions are not reducible to each other. Thus, different forms of checking are independent not only in terms of the specific intentions, but also in terms of the sensitivity condition. Moreover, I provide in Chapter 4 an account of checking de dicto and checking de re. I also distinguish between checking that *x* is F (and that not something else is F) and checking that *x* is F (and that *x* is not something else), and I investigate checking and wh-clauses, for example, checking who was *F*, where *E* happened, or why *E* happened.

The second part of Chapter 4 is devoted to checking and discrimination. There are obvious differences between checking and the capacity of discriminating. Checking is intentional and involves raising a question and intentionally using a method for settling it. Discrimination, in contrast, is not intentional in this sense. Despite these internalistic differences, there are strong externalist analogies. As I argue, the capacity of discrimination via a particular method is best characterized by the method’s modal profile. Perfect discrimination can be characterized as follows:

- S can perfectly discriminate between x and y via M iff
  1. In the nearest possible worlds where the object in question is x and S uses M, M indicates to S that it is x (and not y).
  2. In the nearest possible worlds where the object in question is y and S uses M, M indicates to S that it is y (and not x). (KC, 104)

Thus, perfect discrimination has the same modal profile as perfect checking methods. Moreover, random methods, opposing methods, and monotonous methods are not adequate for discrimination. As I argue, sensitivity is the crucial modal condition on discrimination, i.e., *S* cannot discriminate *x* from *y* via method M if M would indicate that the target item is *x* if it were *y*.^5^

## Chapter 5: Checking, Inferences, and Necessities

Nozick not only provided an account of knowledge in general but also the following account of inferential knowledge.

- S knows (via inference from *p*) that *q* iff
  1. S knows that *p*.
  2. *q* is true, and S infers *q* from *p* (thereby, being led to believe that *q*).
  3. if *q* were false, S wouldn’t believe that *p* (or S wouldn’t infer *q* from *p*).
  4. if *q* were true, S would believe that *p* (and would infer *q* from *p* if she were to infer either *q* or ~ *q* from it). (Nozick 1981, 233f)

In the first part of Chapter 5, I investigate Nozick’s account of inferential knowledge. Knowledge is not closed under known entailment, according to Nozick’s general knowledge account, and we acquire the same results for a deduction. Some instances of deduction fulfill the modified sensitivity condition (3) for inferential knowledge while others do not. Moreover, I argue, against orthodoxy, that some instances of induction are insensitive but others are sensitive according to Nozick’s account of inferential knowledge.^6^ Furthermore, it allows for acquiring abductive knowledge via *the* best explanation.

The second part of Chapter 5 applies the considerations on inferential knowledge to checking. When checking, we intentionally use a method for settling a question. This can also involve inferences. The methods of inferential checking can be specified in a narrow sense as consulting the very premises, or in a wide sense as consulting the sources that delivered the premises. If specified in a narrow sense, then the method is trivially insensitive. Thus, only the wide specification is acceptable. This wide specification of inferential checking delivers the same result as Nozick’s account of inferential knowledge. Some instances of deduction can be methods of checking while others are not, perhaps a controversial result. Furthermore, some instances of induction are sensitive and therefore can constitute checking, while others are not, perhaps a surprising view about the sensitivity of induction, and inference to the best explanation can be checking methods.

Part 3 of Chapter 5 is devoted to problems concerning knowing and checking necessities. Logical necessities are a tricky terrain for modal accounts of knowing and checking because beliefs in necessities trivially or vacuously fulfill the modal conditions of sensitivity and safety. This has the implausible consequence that any belief in a logical necessity constitutes knowledge, or that any method is an instance of checking according to sensitivity accounts or safety accounts. This problem is well known. Nozick (1981) suggests dropping the sensitivity condition for logical necessities and only requiring fulfillment of the belief condition, the truth condition, and the adherence condition. Pritchard (2009) suggests understanding safety not only as a condition on a single proposition but as a broader condition concerning a class of propositions such that beliefs in necessary truths can be unsafe. These problems also affect the modal account of checking. In KC, I propose an alternative solution. I use Nolan’s (1997) account of impossible worlds for counterpossibles (counterfactuals with impossible antecedents) and argue that, for determining sensitivity and safety, we should also take impossible worlds into account. According to this account, some methods are sensitive or safe concerning a necessary truth while others are not, and, consequently, some methods count as methods for checking necessities, while others do not.^7^

## Chapter 6: SAC and Knowledge Puzzles

The first part of KC develops a theory about checking. The second part explains persistent knowledge puzzles in terms of intuitions about checking. In Chapter 6, I explain closure puzzles, centering on lottery cases and deception cases. The explanation that KC provides relies on an assumption about the connection between intuitions about checking and intuitions about knowing. The main connection is expressed by the following principle KSAC:

- KSAC
  - In contexts of checking, when we raise the question whether *p* (or an alternative *q*) is true and deliberate about methods for settling this question, we tend to think that we do not know that *p* via strongly insensitive methods, especially not via monotonous methods. In other contexts, this tendency does not apply. (KC, 142)

KSAC is a principle about knowledge intuitions. It remains neutral about whether these intuitions are true or false. KSAC, fully applied, is not only a principle about self-ascriptions. It also includes intuitions about others who we think are in checking contexts. KSAC can provide two types of explanations for knowledge intuitions. First, it explains when we enter a checking context, and, second, it holds that, in checking contexts, we regard methods as defective for acquiring knowledge that are not checking methods. In particular, we regard strongly insensitive methods as defective.

KSAC can explain some but not all low-stakes/high-stakes puzzles. However, its main application is closure puzzles. Closure puzzles arise when we have conflicting intuitions of knowing an ordinary proposition *o*, but not knowing a proposition *p*, despite knowing that *o* entails *p*. We can distinguish two types of closure puzzles. First, there are closure puzzles involving deception propositions, *d*. Second, there are closure puzzles involving lottery propositions, *l*. In both cases, we intuitively know the first, ordinary proposition, but not the second proposition even though we know that the first entails the second.

Importantly, deception propositions and lottery propositions are both paradigmatically insensitively believed. Based on this insight, KSAC can be used to explain closure puzzles. In the contexts of checking whether *d*/*l* is true, we tend to think that we do not know *d*/*l* since the evidence for *d* or *l* is insensitive.^8^ However, the evidence for the ordinary proposition *o* is sensitive. Therefore, we tend to think in contexts of checking that we know that *o*. Thus, we have, in terms of sensitivity, an explanation of our conflicting intuitions of knowing ordinary propositions but not knowing deception propositions or lottery propositions.

Various solutions to closure puzzles are proposed in the literature. Strict invariantism is the view that we neither know *o* nor *d*/*l*. Moderate invariantism holds that we know both. Ascriber contextualism argues that the standards for knowledge depend on the interests of the knowledge ascriber. When the standards are low, it is true to assert that we know both, but when the standards are high, it is true to assert that we know neither. Subject-sensitive invariantism provides a similar analysis, but it is instead the practical interests of the believing subject, not of the knowledge ascriber, that alter what is known. Finally, one can reject knowledge closure and assume that we know *o* but do not know *d*/*l*. In Chapter 6, I analyze how the KSAC-based explanation of closure puzzles can support these solutions, while remaining neutral about what the correct solution to closure puzzles is. Finally, I compare the KSAC-based explanation to alternative explanations of closure puzzles and argue for the superiority of the explanation provided in KC.

## Chapter 7: Checking and Bootstrapping

Chapter 7 investigates whether bootstrapping is a method of checking. Bootstrapping, as introduced by Vogel (2000), is the process of reasoning about the reliability of a source via information delivered by that very source, an intuitively defective reasoning process. Bootstrapping delivers the result that the target source is reliable regardless of whether this is true or false. Therefore, bootstrapping is a monotonous method and strongly insensitive. Thus, we cannot check via bootstrapping that a source is reliable. As I argue, this holds for standard forms of bootstrapping via induction but also for deductive bootstrapping. This leads to limitations of the possibilities of checking the reliability of a source. I argue that, for checking the reliability of a source, we have to use another source. Moreover, this other source must be modally independent from the target source. We acquire similar results for checking whether a particular indication of a source is true. Again, we cannot check whether O’s indication that *p* is true by using an alternative source O’ with the same modal profile as O.

The second part of Chapter 7 is devoted to intuitions about knowing via bootstrapping. The fact that bootstrapping is, due to its insensitivity, an inadequate method for checking also provides an explanation of our intuitions that it is a flawed method for acquiring knowledge. The full explanation of our intuitions that we do not know via bootstrapping developed in KC is as follows:

When deliberating about bootstrapping, we tend to enter a context of checking for two reasons: First, because we reason about the reliability (or accuracy) of a source; second, because bootstrapping resembles paradigmatic processes of checking or testing a source’s reliability or accuracy, but in a caricaturing way. In contexts of checking, we regard methods of which we think that they are strongly insensitive as inadequate for acquiring knowledge. The monotonicity and, therefore, strong insensitivity of bootstrapping is obvious for us. Therefore, we regard bootstrapping as an epistemically flawed method. Consequently, we think that we cannot *know* that a source is reliable or accurate via bootstrapping. (KC, 207)

Again, I focus on explaining knowledge intuitions, leaving open at this point whether our negative intuitions about knowing via bootstrapping are true.

## Chapter 8 SAC and the Skeptical Puzzle

The final chapter of KC is devoted to explaining and solving the skeptical puzzle. In the first part, I review the skeptical problem and various solutions that have been proposed. The skeptical puzzle can best be understood as a puzzle about three conflicting intuitions, each intuitively appealing but jointly inconsistent: (1) that we have knowledge of the external world, (2) that we do not know that the skeptical hypothesis, ~ *sh*, is false, and, (3) if we have knowledge of the external world, then we know that ~ *sh*. The skeptic accepts (2) and (3) and rejects (1), while Mooreanism endorses (1) and (3) and rejects (2). Concerning Mooreanism, I identify in KC a further puzzle constituted by two conflicting intuitions:

- The Moorean puzzle
  - MI_1_: Moorean reasoning is flawless. It is in line with the commonsensical and philosophically popular view that perceptual knowledge can be immediate, and it is in line with ordinary reasoning about the external world, about our own mental states and about their accuracy.
  - MI_2_: Moorean reasoning is flawed because it can only deliver the result that skeptical hypotheses are false. (KC, 220)

The second part of Chapter 8 is devoted to explaining the skeptical puzzle and the Moorean puzzle. First, I reflect on doubting and checking whether one’s own belief that *p* is true, which is distinct (also in terms of sensitivity) from doubting and checking whether *p* is true. I explain why various forms of bootstrapping and Moorean reasoning, due to their insensitivity, fail to be instances of checking whether one’s own beliefs are true. Based on these insights about problems and limits of checking whether one’s own beliefs are true, I provide the following explanation of the Moorean puzzle:

Doubting one’s own beliefs is typically the first step of a process of checking of one’s own beliefs whether they are true. In such checking contexts, we tend to think that we cannot know that our beliefs are true via strongly insensitive methods, especially not via monotonous methods. Moorean reasoning is obviously a monotonous method, always indicating that the checked beliefs are true regardless of whether they are actually true. Therefore, in skeptical contexts of doubting, we think that we do not know via Moorean reasoning that our beliefs are true and that Moorean reasoning is a flawed response to the skeptical challenge. In contexts of ordinary self-reflection, this tendency does not apply. In these contexts, we think that we have immediate external world knowledge and knowledge that our sense apparati are reliable and that the skeptical hypothesis is false in a way that is a complex form of Moorean reasoning. (KC 233f)

This explanation leaves open which of our conflicting intuitions about Moorean reasoning are true. Accordingly, it is compatible with positive and negative views about knowledge via Moorean reasoning. As I contend, we have to distinguish two cognitive processes of higher-level reflection, checking whether one’s beliefs are true and ordinary self-reflection, even if both processes involve the same propositions. Ordinary self-reflection about the truth of our own beliefs involves a complex form of Moorean reasoning. I suggest that Moorean reasoning is a form of acquiring knowledge that ~ *sh*, but it is not a form of checking. This view is compatible with Mooreanism but also with checking-based versions of contextualism and subject-sensitive invariantism.

In the final part of Chapter 8, I address the heterogeneity problem of sensitivity that I introduced in Melchior (2015). According to this problem, sensitivity delivers concerning higher-level knowledge about the truth or falsity of one’s own beliefs a picture too heterogeneous to be plausible. As I argue, the provided account of checking does not face this problem, because checking *of one’s beliefs whether they are true* delivers a homogenous picture, since all instances of Moorean reasoning are insensitive and, therefore, fail to be methods of checking of one’s own beliefs whether they are true or not false. Moreover, the account provided in KC avoids a version of the generality problem for Moorean reasoning, as presented in Melchior (2014b).

## Outlook

The provided sensitivity account of checking is an open project with further applications. These applications include, for example, a sensitivity-based account of medical testing and a sensitivity-based account of why consulting statistical evidence fails as a method of checking with applications to proof in law.

## Footnotes

1 These views about the insensitivity of inductive beliefs and higher-level beliefs did not remain uncontested. For the view that induction leads to sensitive beliefs, see Wallbridge (2018), and for a more nuanced picture, see Melchior (forthcoming). For the view that the crucial higher-level beliefs are sensitive, see Becker (2006 and 2007) and Salerno (2010), and for the view that the (in)sensitivity of higher-level beliefs delivers a picture too heterogeneous to be plausible, see Melchior (2015). For an objection against this view and a reply, see Wallbridge (2017) and Melchior (2018).

2 For a criticism of DeRose’s sensitivity account, see Melchior (2014a).

3 Negative safety depends on what would be the case if method M were indicating that *p* is *false*.

4 I assume here that the subject does not know that the method is opposing.

5 A more detailed sensitivity account of discrimination is developed in Melchior (2021a).

6 For a detailed investigation of the sensitivity of induction, see Melchior (forthcoming).

7 For a more detailed impossible world account for sensitivity and safety, see Melchior (2021b). For a discussion of alternative accounts, see Melchior (2017).

8 This tendency also applies in contexts of checking whether *d*/*l or o* is true.
